# Assessing Loggerhead Turtle Exposure to Fisheries in Northwest Africa: Predicted Risk and Management Gaps

**DOI:** 10.1002/ece3.73729

**Published:** 2026-06-01

**Authors:** Amy Isabelle Bowler, Annette C. Broderick, Michael S. Coyne, Matthew H. Godfrey, Brendan J. Godley, Pedro Lopez‐Suarez, Nuria Varo‐Cruz, Lucy. A. Hawkes

**Affiliations:** ^1^ School of Biosciences, Faculty of Health and Life Sciences University of Exeter Exeter UK; ^2^ Centre for Ecology and Conservation, Faculty of Environment, Science and Economy University of Exeter Penryn UK; ^3^ Seaturtle.org Durham North Carolina USA; ^4^ North Carolina Wildlife Resources Commission Raleigh North Carolina USA; ^5^ SharMarine Science Research Centre University of Khorfakkan Sharjah UAE; ^6^ BIOS.CV Association for Environmental Conservation and Sustainable Development Sal Rei Cabo Verde; ^7^ Cetaceans and Marine Research Institute of the Canary Islands CEAMAR Las Palmas Spain

**Keywords:** biologging, bycatch, fisheries management, overlap

## Abstract

Bycatch is the accidental capture of non‐target animals in fishing gear, and is a critical threat to many wildlife populations, impeding recovery and conservation efforts. Northwest Africa hosts a significant population of loggerhead turtles ( *Caretta caretta* ), particularly around Cabo Verde, which is among the world's top three largest loggerhead turtle nesting colonies. However, high‐risk areas for turtle bycatch have not yet been comprehensively investigated. This study addressed this by analysing biologging data from loggerhead turtles (*n* = 26), quantifying their spatial and vertical overlap with fishing in the Northeast Atlantic. Results revealed extensive overlap of loggerhead turtles with fishing across seven countries, with particularly intense overlap in Cabo Verde, Senegal and Mauritania. Turtles also generally occupied the same depths as fishing gear, intensifying bycatch risk. Among fishing methods, trawling showed the greatest overlap with loggerhead turtles, and current protections appear to align poorly with our predictions of bycatch risk. Coupled with increasing fishing pressure, these findings highlight the need for strengthened conservation measures. These include gear modifications such as turtle excluder devices, as well as time and space‐based fisheries management strategies. Going forward, improved bycatch reporting and monitoring across all fisheries sectors will be essential to expand upon these findings.

## Graphical abstract

Biologging and Global Fishing Watch data reveal extensive spatial and vertical overlap between loggerhead turtles from the Cabo Verde population and fisheries across the Northeast Atlantic, highlighting areas of high predicted bycatch risk and gaps in current protections.

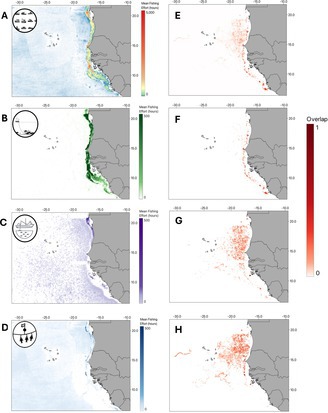

## 1 Introduction

Marine species face a wide range of anthropogenic threats, including climate change, pollution, habitat destruction, and overfishing, which are intensifying globally and are expected to double in cumulative impact by mid‐century (O'Hara et al. 2021; Halpern et al. 2015, 2025), driving declines across marine fauna (McCauley et al. 2015). Among these threats, fishing is one of the most pervasive and impactful human activities on marine ecosystems (Halpern et al. 2008, 2025; Paolo et al. 2024). It has led to the overexploitation of a third of the world's commercial fish and invertebrate stocks (FAO 2024) while also endangering non‐target species through resource competition (Jusufovski et al. 2019), vessel collisions (Schoeman et al. 2020), and incidental capture or ‘bycatch’ (Clay et al. 2019). Entanglement in fishing gear can cause injuries, physiological stress, or mortality (Wilson et al. 2014), and bycatch is the primary driver of population declines in several species, including sea turtles (Wallace et al. 2013), seabirds (Clay et al. 2019), and cetaceans (Rolland et al. 2016). The loss of marine megafauna due to bycatch has significant ecological consequences, as these species play key roles in maintaining ecosystem stability, connectivity, and biogeochemical cycling (Estes et al. 2016). Furthermore, despite stabilising or declining global catches, fishing power and effort have increased disproportionately over time, making the global scale and intensity of fishing an escalating threat to marine life (Watson et al. 2013; Bell et al. 2017).

Until recently, the global scope of fishing activities was poorly understood. Traditional monitoring methods, such as automated tracking of large vessels by their flag state, or self‐reported data from fishing boats, have limitations regarding accessibility and reliability (Dunn et al. 2018). The introduction of Global Fishing Watch (GFW) in 2018 transformed this by providing a global and dynamic record of fishing on a publicly accessible platform (Kroodsma et al. 2018). GFW uses Automatic Identification System (AIS) data, originally developed as a collision‐avoidance tool (McCauley et al. 2016). AIS devices on vessels transmit their location and movement, and GFW applies machine learning to these data to distinguish fishing from transiting, as well as different types of fishing, based on speed and direction. The resulting dataset offers spatial and temporal detail exceeding previous monitoring methods by several orders of magnitude and tracks 50%–75% of vessels over 24 m in length worldwide. However, AIS are only legally mandated on larger industrial vessels, meaning small‐scale and artisanal fisheries are often absent and under‐represented in GFW data (Kroodsma et al. 2018).

Resolving the threat of bycatch to marine organisms requires identifying areas where species' movements overlap with fishing (Lewison et al. 2014). Such overlap studies have been conducted for seabirds, elasmobranchs, and sea turtles, helping to identify bycatch risk hotspots (Araújo et al. 2022; Queiroz et al. 2019; Almpanidou et al. 2022). Vertical overlap—how species and fishing gear occupy similar depths—has been incorporated into fewer studies (Barbour et al. 2023). Insights from overlap studies are essential for evaluating current conservation measures and guiding new, targeted management strategies (Giménez et al. 2021; Klimley et al. 2022; Santos et al. 2019).

Loggerhead turtles ( *Caretta caretta* ) are globally distributed across subtropical and temperate waters (Wallace et al. 2010). Cabo Verde, an archipelago approximately 500 km off the West African coast, hosts one of the world's largest nesting populations; while earlier estimates reported approximately 15,300 nests annually (Marco et al. 2012), more recent island specific assessments indicate substantially higher nesting numbers (Laloë et al. 2019; Hays et al. 2022). For example, Sal island recorded 35,507 nests in 2020 (Hays et al. 2022). These turtles appear to be genetically distinct from other Atlantic and Mediterranean populations and are managed as a separate Northeast Atlantic Regional Management Unit (Wallace et al. 2010). The population is classified as endangered (Marco and Casale 2015), despite the increase in nesting numbers over the last decade (Hays et al. 2022). A recent re‐evaluation of turtle management units has also improved the population's risk score (Wallace et al. 2025). However, significant concerns remain. As seen in other recovering turtle populations, current nesting figures likely represent only a small fraction of historical abundance (McClenachan et al. 2006), and substantial data gaps hinder a complete assessment of recovery (Wallace et al. 2025).

The wider Northeast Atlantic loggerhead population faces multiple threats, including direct harvesting of turtles and eggs (Hancock et al. 2017), hatchling misorientation from light pollution (Taylor and Cozens 2010), and skewed sex ratios due to climate change (Laloë et al. 2017). However, bycatch is still likely the most significant threat to this population (Riskas and Tiwari 2013). Northwest African waters are among the world's most productive fishing grounds due to upwelling systems, resulting in high fishing pressure (Ohde and Siegel 2010; Jamal et al. 2024). The combination of intense fishing activity and high turtle densities may therefore make the region a global hotspot for turtle bycatch (de la Hoz Schilling et al. 2023; Cardona et al. 2025). It is estimated that tens of thousands of turtles may be caught annually in Northwest Africa, and loggerheads are one of the most commonly caught species in the region (de la Hoz Schilling et al. 2023; Cardona et al. 2025). For example, Cardona et al. (2025) estimate that 6800 loggerhead turtles are killed each year by longline fishing alone in the Canary Current Large Marine Ecosystem. Such mortality levels are concerning, as population models have predicted, despite the observed increase in nesting numbers (which may be driven by primary productivity or climate change), population declines if current bycatch levels persist (Roast et al. 2023; Nugraha et al. 2026).

The present study quantifies fisheries threat to turtles from the Northeast Atlantic loggerhead population, with three specific objectives: (1) to use biologging data and GFW data to map loggerhead turtle movements and fishing activity throughout the region; (2) to assess horizontal and vertical overlap between turtles and fisheries; and (3) to evaluate current conservation measures and propose targeted regional fisheries management strategies to mitigate bycatch risk more effectively.

## 2 Methods

### 2.1 Turtle Movements and Fishing Data

This study used data from 26 loggerhead turtles tracked with satellite‐linked transmitters in the Northeast Atlantic between 2004 and 2006. The turtles were all adult females and were tagged after laying nests on beaches in Cape Verde. Locations were transmitted via the ARGOS system, and locations with low spatial accuracy (classes Z and 0), were excluded (i.e., retaining classes 3, 2, 1, A and B) as well as locations on land, and locations implying unrealistic movement between successive fixes (> 1.5 km/h, > 5000 m, or acute angles (< 25°) between any three consecutive locations). The resulting dataset included 13,142 locations with which density rasters were generated with a 0.1° × 0.1° resolution by summing locations within each cell. Foraging strategies were classified as neritic or oceanic, following Hawkes et al. (2006).

Fishing effort data (2013–23) from GFW (https://globalfishingwatch.org) were obtained and processed using the gfwr package in R. Data described fishing hours per grid cell, and were extracted at a 0.1° × 0.1° resolution, and then grouped by Exclusive Economic Zone (EEZ) and flag state. Fishing effort was also categorised by gear type, focusing on longlines, trawlers, and purse seines, as these likely pose the greatest bycatch risk (Alessandro and Antonello 2010; Bourjea et al. 2014; de la Hoz Schilling et al. 2023). Small‐scale and artisanal fisheries are not included in GFW data as they do not legally have to carry AIS, despite their prevalence in Northwest Africa (Belhabib et al. 2018).

Turtle tracking data (2004–2006) preceded the fishing effort dataset (2013–2023) by approximately 10 years. While this temporal mismatch introduces some uncertainty, particularly as climate‐driven changes in ocean conditions may alter turtle and prey distributions, both loggerhead distributions and fishing effort in the Northeast Atlantic are structured by persistent oceanographic features, such as regional upwelling systems, and may therefore concentrate in broadly similar areas over time. Importantly, overlap estimates are interpreted as indicators of relative spatial risk rather than absolute contemporary exposure.

### 2.2 Overlap

Spatial overlap between turtle distributions and fishing effort was quantified using Bhattacharyya's coefficient, a measure of co‐occurrence in each grid cell (Bhattacharyya 1943; Hatch et al. 2023). This analysis assessed spatial overlap only and did not explicitly incorporate temporal or seasonal variation in overlap. To ensure comparability and equal contribution to the overlap index while preserving magnitude differences, turtle and fishing effort rasters (0.1° resolution) were rescaled to a 0–1 range:

$$R_{\text{rescaled}}=\frac{R-\min\left(R\right)}{\max\left(R\right)-\min\left(R\right)}$$

where $R_{\text{rescaled}}$ = Rescaled raster value, R = Original raster value, and $\max\left(R\right)$, $\min\left(R\right)$ = Maximum and minimum values of the original raster. Bhattacharyya's coefficient ($B_{c}$) was calculated for each grid cell as:

$$B_{c}=\sqrt{R_{\text{fishing},\text{rescaled}}\times R_{\text{turtle},\text{rescaled}}}$$

where $R_{\text{fishing},\text{rescaled}}$ = Rescaled fishing effort raster, and $R_{\text{turtle},\text{rescaled}}$ = Rescaled turtle density raster. Finally, $B_{c}$ was normalised to span the complete range from 0 (no overlap) to 1 (maximum overlap), to produce the overlap coefficient (O).

$$O=\frac{B_{c}}{\max\left(B_{c}\right)}$$

### 2.3 Statistical Analyses

Fishing effort for each gear type was extracted at turtle locations from the corresponding gear‐specific fishing effort raster, and differences in overlap with turtles among gear types were tested using a Kruskal‐Wallis test, followed by Dunn's test for pairwise comparisons to account for non‐normality. EEZ polygons were obtained from the Maritime Boundaries Geodatabase (Version 12; doi:10.14284/632). The sum of overlap coefficients within each EEZ polygon was calculated using the zonal statistics tool in QGIS and expressed as a proportion of the total overlap across all EEZs. Fishing effort values were also extracted separately for neritic and oceanic turtle locations, and a Mann–Whitney *U* test was used to compare the extent of overlap. Fishing effort for each gear was also compared between the two turtle groups using separate Mann–Whitney *U* tests. Within each turtle group, gear types were also compared; pairwise comparisons were conducted using Mann–Whitney *U* tests with Holm's correction to account for multiple comparisons.

### 2.4 Vertical Overlap

Dive data were obtained from depth‐recording tags deployed on two loggerhead turtles. As dive behaviour may vary among individuals and life stages, these data should be considered illustrative of potential vertical overlap with fisheries rather than representative of the wider population. High‐fishing‐effort grid cells (where fishing effort was greater than the overall mean across turtle locations) were selected for each gear type and retained for analysis if one of the turtles travelled through it. Fishing gear deployment depths were estimated from literature sources (NOAA Fisheries 2025; Jacquemont et al. 2024; Bigelow et al. 2006; Puig et al. 2012) and compared with turtle maximum dive depth and seabed depth (from GEBCO bathymetry data).

### 2.5 Conservation

Marine Protected Area (MPA) shapefiles were obtained from the World Database on Protected Areas (UNEP‐WCMC 2025). Fishing inside MPAs was quantified by summing fishing hours within MPA boundaries. Current bycatch mitigation measures in each EEZ were reviewed using reports from the 3rd Meeting of Signatories of the Atlantic Turtle Memorandum of Understanding (CMS MoU) (CMS 2023). A conservation score was assigned to each EEZ based on the presence and implementation of mitigation measures, with scores ranging from 0 (no measure) to 3 (high implementation) for each measure. Emerging sea turtle bycatch mitigation strategies were reviewed through literature and the Conservation Evidence database (Sainsbury et al. 2021).

## 3 Results

### 3.1 Turtle Movements

**FIGURE 1:**
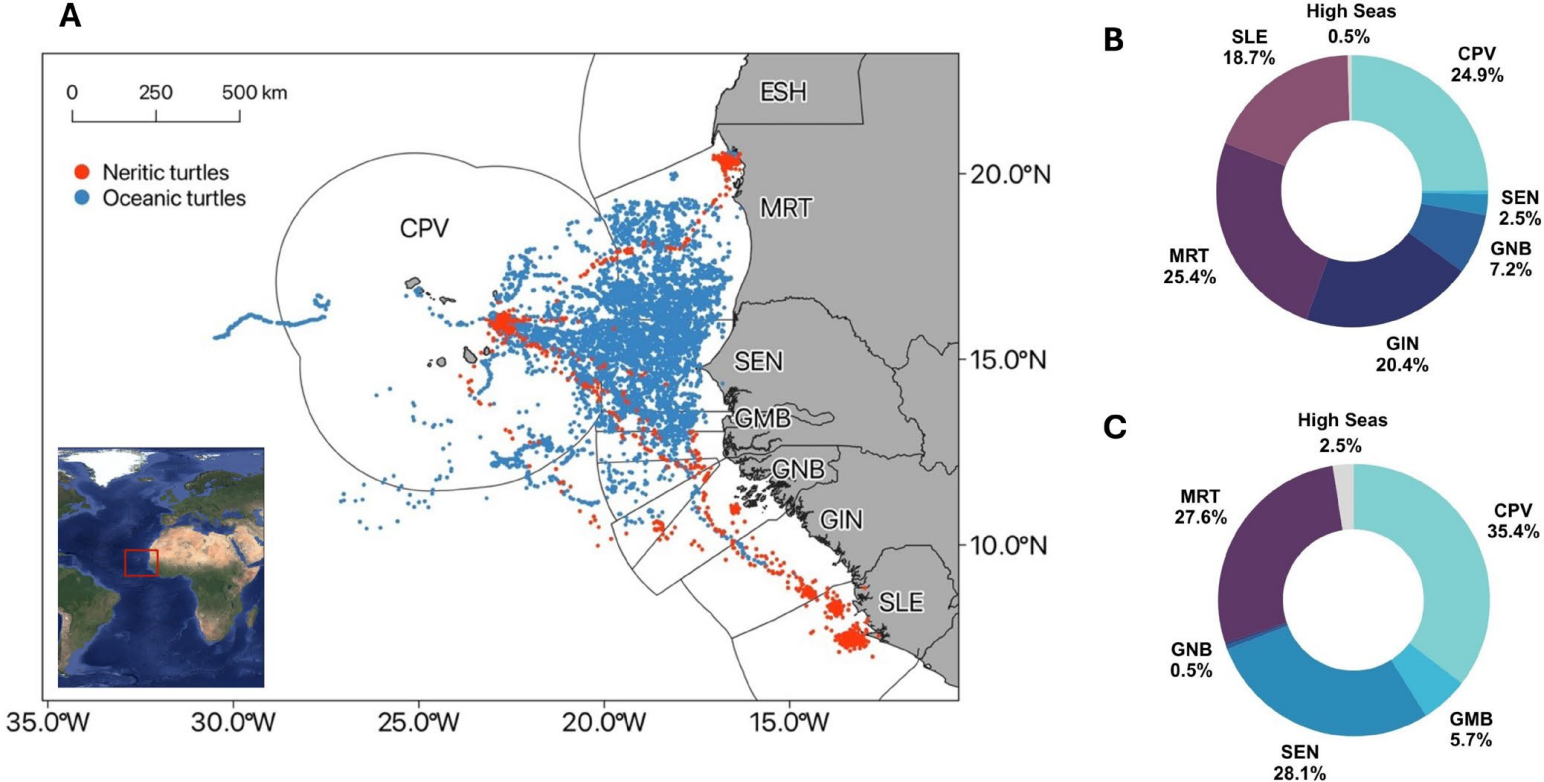
Map showing the distribution of neritic (red) and oceanic (blue) turtle locations recorded from satellite‐transmitting tags (A). Donut plots showing the proportion of locations within each Exclusive Economic Zone (EEZ) for neritic (B) and oceanic (C) turtles (CPV = Cabo Verde, ESH = Western Sahara, GIN = Guinea, GMB = The Gambia, GNB = Guinea‐Bissau, MRT = Mauritania, SEN = Senegal, SLE = Sierra Leone).

Loggerhead turtles exhibited extensive movements across a total area of approximately 1.7 million km^2^. Movements spanned seven EEZs, with 2% of locations in international waters (Figure 1A). The highest proportions of turtle locations were in Cabo Verde (33.2%), followed by Mauritania (27.2%), Senegal (22.7%), The Gambia (4.6%), Guinea (4.5%), Sierra Leone (3.9%), and Guinea Bissau (1.9%). Neritic turtles (*n* = 7), which moved directly to relatively localised coastal foraging grounds, were most often found in the EEZs of Mauritania, Cabo Verde and Guinea (Figure 1B), while oceanic turtles (*n* = 19), which made extensive, wide‐ranging movements throughout the tracking period, were most often found in the EEZs of Cabo Verde, Senegal and Mauritania (Figure 1C).

### 3.2 Fishing Effort

**FIGURE 2:**
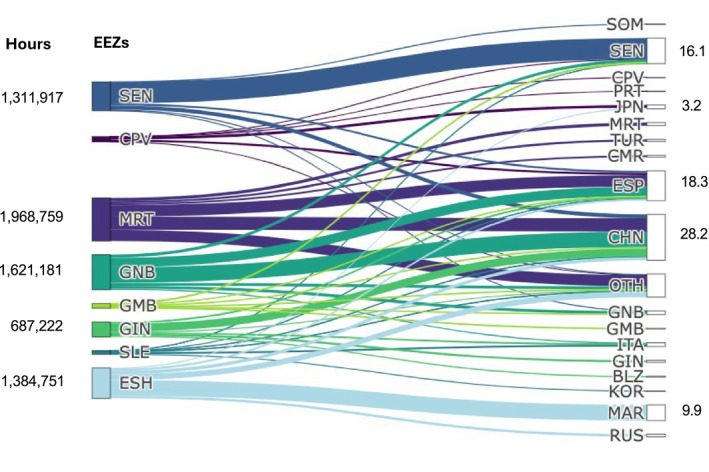
Sankey diagram illustrating the distribution of fishing effort across Exclusive Economic Zones (EEZs), the flag states of vessels fishing in them, and the connections, the thickness of which is proportional to total fishing hours (2013–2023). Total fishing hours for the top five EEZs are shown on the left, and the percentage of total effort for the top five flag states is shown on the right (BLZ = Belize, CHN = China, CMR = Cameroon, CPV = Cabo Verde, ESH = Western Sahara, ESP = Spain, GIN = Guinea, GMB = The Gambia, GNB = Guinea‐Bissau, ITA = Italy, JPN = Japan, KOR = South Korea, MAR = Morocco, MRT = Mauritania, OTH = Other, PRT = Portugal, RUS = Russia, SEN = Senegal, SLE = Sierra Leone, SOM = Somalia, TUR = Turkey).

**FIGURE 3:**
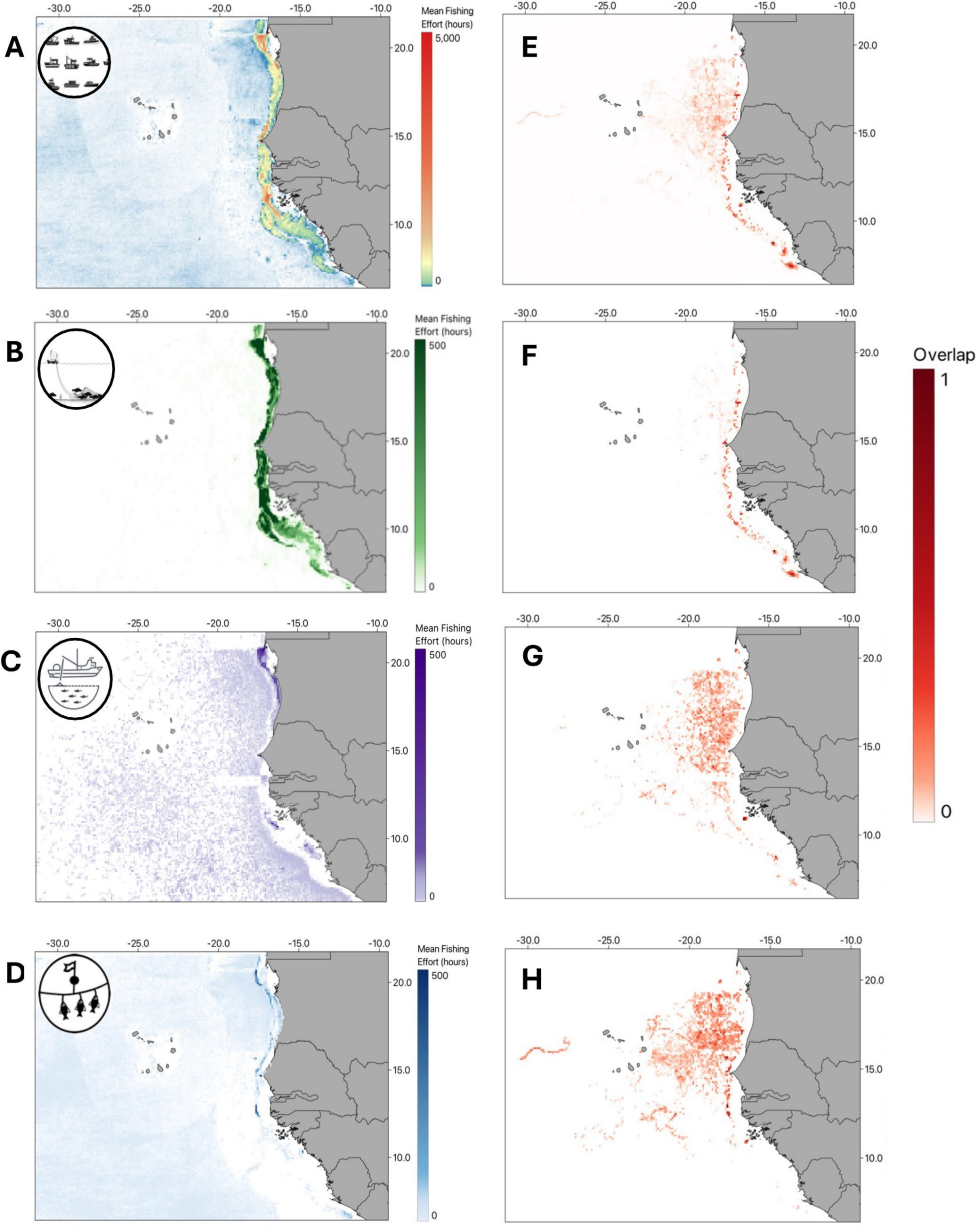
Spatial distribution of fishing effort and overlap with turtle locations by gear type. Panels (A–D) show annual mean fishing effort (2013–23) for (A) all gear types combined, (B) trawling, (C) purse seines, and (D) longlines. Panels (E–H) display the spatial overlap between turtles and fishing effort, for (E) all gear types combined, (F) trawling, (G) purse seines, and (H) longlines. The overlap coefficient (0–1) is represented by varying intensities of red, where higher values indicate greater overlap. Note that overlap was normalised separately for each gear type; therefore, colour intensity reflects relative overlap within each panel and is not directly comparable between panels.

Fishing vessels undertook 7,510,485 h of fishing in the study area from 2013 to 2023. Total fishing effort was highest in Mauritania (1,968,759 h), followed by Guinea‐Bissau (1,621,181 h), and Western Sahara (1,384,751 h) (Figure 2). Vessels from 74 nations operated across the eight EEZs analysed, with the greatest effort from China (28.2%), Spain (18.3%), and Senegal (16.1%) (Figure 2). Fishing effort was widespread but more concentrated along the coast (Figure 3A). Trawlers accounted for 63.7% of fishing hours, followed by longlines (17.6%), and purse seines (4.4%). Other gear types included fixed gear, pole and line, and gillnets. These reflect AIS‐detectable industrial fleets and do not include small‐scale, artisanal fisheries, which are widespread in the region but not spatially resolved in GFW data. Trawling was concentrated along the coastline (Figure 3B), but purse seines were widespread, with a hotspot off northern Mauritania (Figure 3C), and longlines were similarly widespread throughout northern EEZs including Mauritania (Figure 3D).

### 3.3 Overlap

The majority (mean 79% overall; median 80%, range 0%–100% per turtle) of turtle locations occurred within areas where there was at least some fishing during the study period. Overlap was widespread (Figure 3E), with hotspots along the African coastline that varied by fishing type. Trawling overlap was particularly concentrated along the coastline (Figure 3F), while purse seine overlap was more widespread across oceanic regions, primarily northeast of Cabo Verde, within the Mauritanian and Senegalese EEZs (Figure 3G). Longline overlap was also widespread across oceanic regions and highest in Mauritania, Senegal, and The Gambia (Figure 3H).

**FIGURE 4:**
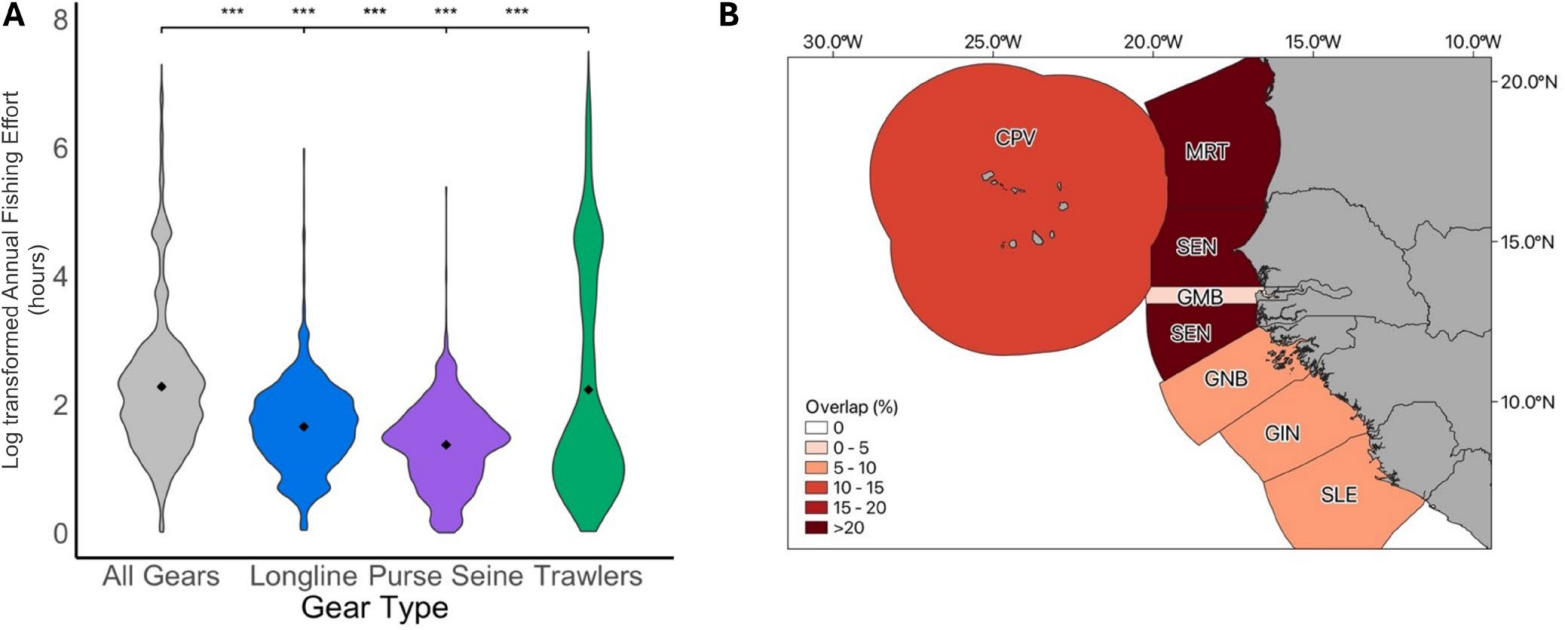
Overlap by Gear type and EEZs. (A) Log‐transformed annual fishing effort at turtle locations, categorised by fishing gear type. The mean annual fishing effort for each gear type is represented by a black point. *p* < 0.001 significance levels are denoted by ***. (B) Map illustrating the proportion of overlap occurring within each Exclusive Economic Zone (EEZ), calculated as a percentage of the total overlap across the study region.

**FIGURE 5:**
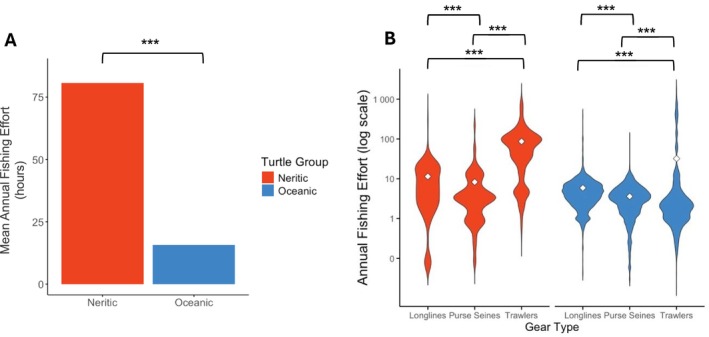
Oceanic and Neritic Turtles. (A) Mean annual fishing effort at neritic and oceanic turtle locations. (B) Annual fishing effort at turtle locations, comparing gear types within neritic and oceanic turtle groups. Values are shown on a logarithmic y‐axis to aid visualisation, and mean values are indicated by white diamond symbols. Statistical significance is denoted by *** for *p* < 0.001.

Average annual fishing effort at turtle locations was highest for trawlers (50.9 h per year), followed by longlines (6.2 h) and purse seines (4.1 h) (Kruskal‐Wallis test, *H* = 3901, *p* < 0.001; Dunn's test, *p* < 0.001; Figure 4A). Trawling effort varied more than other fishing methods, with peaks at both low and high effort levels (Figure 4A). Overlap occurred in all EEZs, but was highest in Mauritania (30%), Senegal (28%), and Cabo Verde (15%), followed by Sierra Leone (9%), Guinea Bissau (7%), Guinea (6%), and The Gambia (4%; Figure 4B). Neritic turtles were exposed to higher average annual fishing effort (80.6 h/location) than oceanic turtles (15.7 h/location; Mann–Whitney *U* test, *W* = 11,100,427, *p* < 0.001; Figure 5A), and this was consistent across all three gear types (trawling *W* = 2,430,529, *p* < 0.001; longlines *W* = 1,457,192, *p* < 0.001; and purse seines *W* = 2,266,852, *p* < 0.01). Neritic turtles faced the highest average annual fishing effort from trawling (86.9 h/location), followed by longlines (11.4 h/location) and purse seines (8.3 h/location; Mann–Whitney *U* tests, trawling vs. longlines *W* = 362,019, *p* < 0.001; trawling vs. purse seines *W* = 719,539, *p* < 0.001; longlines vs. purse seines *W* = 151,331, *p* < 0.001; Figure 5B). Similarly, oceanic turtles experienced the highest average annual effort from trawling (32.2 h/location), followed by longlines (5.92 h/location) and purse seines (3.6 h/location; Mann–Whitney *U* tests, trawling vs. longlines *W* = 5,055,512, *p* < 0.001; trawling vs. purse seines *W* = 6,010,276, *p* < 0.001; longlines vs. purse seines *W* = 26,867,602, *p* < 0.001; Figure 5B).

### 3.4 Vertical Overlap

**FIGURE 6:**
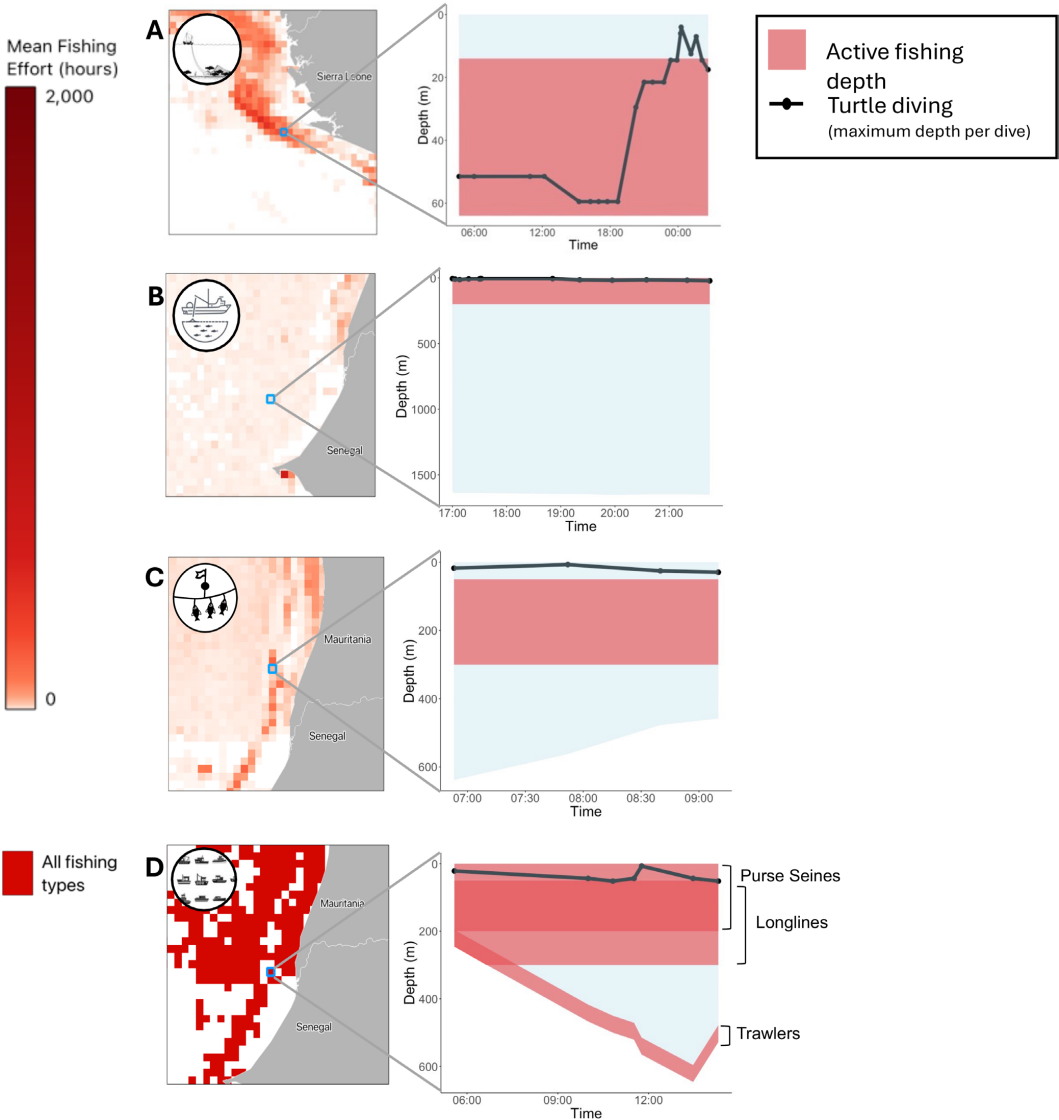
Vertical overlap between fishing gear and turtle diving depths. Annual mean fishing density maps for (A) trawlers, (B) purse seines, (C) longlines, and (D) areas where all three fishing types occur in the same grid cell. For each map, a high fishing intensity grid cell is selected, showing active fishing gear depths (red), maximum turtle dive depths (black points connected with lines), and sea depth (light blue). The maps highlight areas of potential vertical overlap between fishing gear and turtle diving depths.

Estimated active fishing depths ranged from 0 to 200 m for purse seines, 50–300 m for longlines (relative to the sea surface), and up to 50 m above the seabed for trawlers. To illustrate how vertical overlap with turtles may occur, two turtles that were tracked with dive‐recording devices are presented as case studies. Turtle 1, travelling in the Sierra Leone EEZ in January 2006, frequently dived within the estimated active fishing depths of trawlers, doing so on 91% of its dives (Figure 6A). In contrast, Turtle 2, travelling off the coast of Senegal in October 2004, stayed near the surface, within the estimated operating depths of purse seiners (Figure 6B). It also travelled through Mauritania, remaining near the surface and narrowly avoiding the longline zone (Figure 6C). In some areas (Figure 6D) visited by turtles, all three fishing methods were present, and the vertical risk zone thus expanded. In one example, Turtle 2 was in the purse seine risk zone and occasionally approached longline depths.

### 3.5 Conservation

**FIGURE 7:**
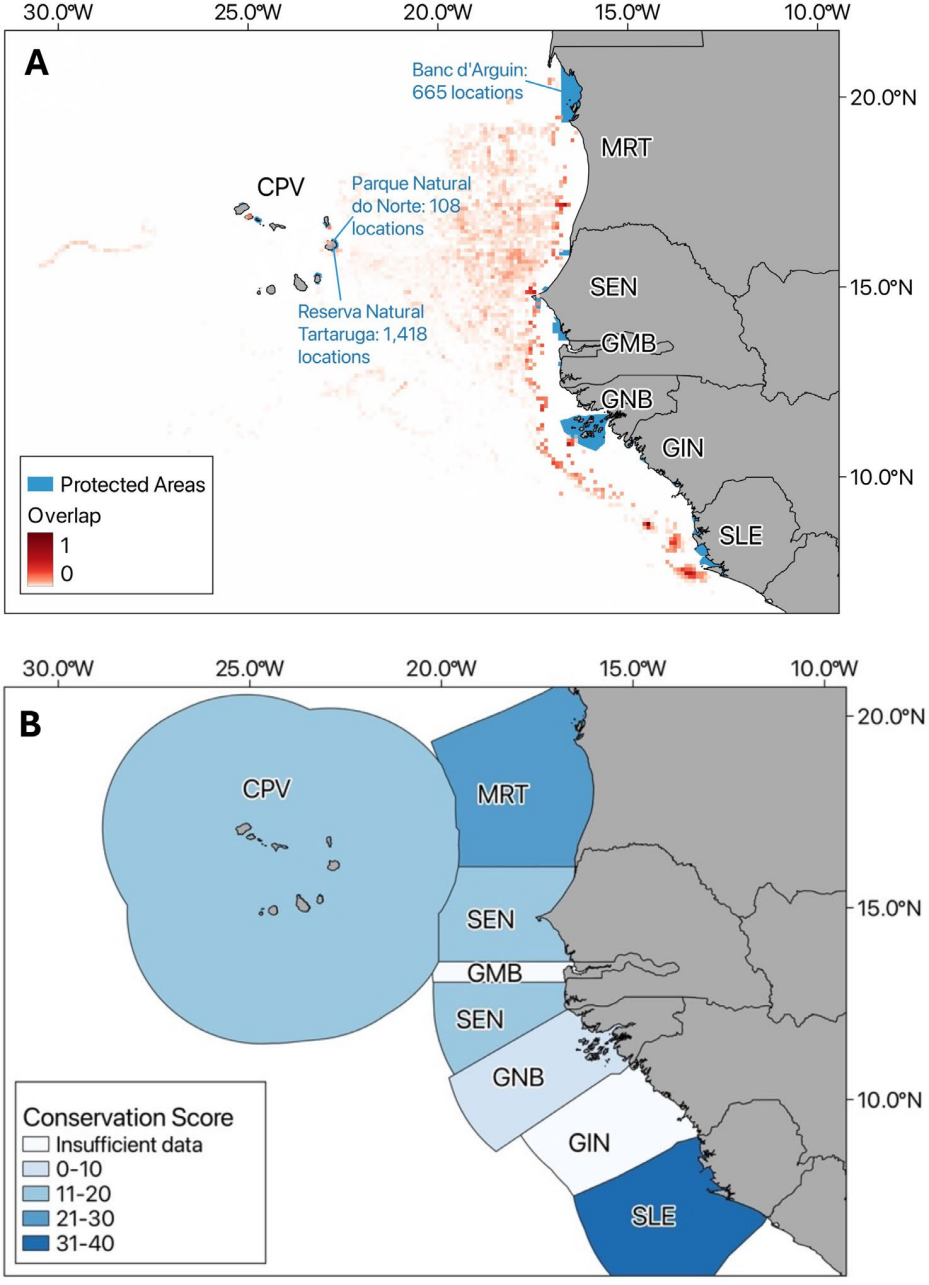
Marine Protected Areas (MPAs) and conservation scores across Exclusive Economic Zones (EEZs). (A) Map showing current protected areas (blue) and bycatch risk hotspots, where fishing overlaps with turtle locations (red). The number of turtle locations within key protected areas is also indicated. (B) EEZs coloured by their conservation score, calculated from reports from the 3rd Meeting of Signatories of the Atlantic Turtle Memorandum of Understanding (CMS MoU).

Turtles overlapped with 11 MPAs, covering 16.9% of tracking locations. The highest numbers were in Reserva Natural Tartaruga MPA, Cabo Verde (*n* = 1418, 10.8% locations), Banc d'Arguin, Mauritania (*n* = 665, 5.1%), and Parque Natural do Norte, Cabo Verde (*n* = 108, 0.8%; Figure 7A). However, fishing activity occurred throughout MPAs in the region, comprising 2.09% of the total fishing effort. Conservation scores calculated from CMS MoU reports were highest for Sierra Leone and lowest for Guinea‐Bissau (Figure 7B and Table 1). Conservation scores could not be assigned to The Gambia or Guinea due to the absence of data. The Conservation Evidence database identified 38 turtle conservation measures, with 14 focused on bycatch, and circle hooks (11 studies), bait type (9 studies), and Turtle Excluder Devices (TEDs) (6 studies) were the most common measures (Table S1).

**TABLE 1:** Conservation measures from CMS MoU reports, listing each country and indicating whether each measure is in place, coloured by implementation level—red for low, yellow for medium, and green for high. N/A indicates no report available. The table also includes each country's overall conservation score, calculated from the listed measures and their implementation levels (CPV = Cape Verde, GIN = Guinea, GMB = The Gambia, GNB = Guinea‐Bissau, MRT = Mauritania, SEN = Senegal, SLE = Sierra Leone).

Bycatch mitigation measure	CPV	GMB	GIN	GNB	MRT	SEN	SLE
Safe handling of incidentally caught turtles (e.g., resuscitation or release using de‐hooking tools, line cutting tools, and scoop nets)	Yes	N/A	N/A	No	Yes	Yes	Yes
Devices that allow the escape of marine turtles (e.g., turtle excluder devices (TEDs))	No	N/A	N/A	No	No	Yes	Yes
Devices that allow marine turtles to avoid the nets (e.g., lights)	No	N/A	N/A	No	No	Yes	No
Measures to avoid encirclement of marine turtles in purse seine fisheries	No	N/A	N/A	No	No	Yes	No
Measures to release the turtles before the purse seine is hauled in	No	N/A	N/A	No	No	Yes	Yes
Appropriate combinations of hook size and design, type of bait, depth, gear specifications, and fishing practices	No	N/A	N/A	No	No	No	Yes
Monitoring and recovery of fish aggregating devices (FADs)	No	N/A	N/A	No	No	No	Yes
Use of eco‐friendly FADs	No	N/A	N/A	No	No	No	Yes
Spatial and temporal control of fishing (e.g., seasonal closures of fishing activities)	No	N/A	N/A	No	Yes	No	No
Onboard observer programmes	No	N/A	N/A	No	Yes	Yes	Yes
Vessel monitoring systems	Yes	N/A	N/A	No	Yes	Yes	Yes
Inspections (i.e., at sea, in port, at landing sites)	Yes	N/A	N/A	No	Yes	Yes	Yes
Law enforcement at sea	Yes	N/A	N/A	No	Yes	Yes	Yes
Training programmes/Workshops to train fishers on the use of bycatch reduction methods	Yes	N/A	N/A	No	Yes	No	Yes
Informative videos, brochures, printed guidelines etc.	Yes	N/A	N/A	No	Yes	Yes	Yes
Proper disposal of discarded gear to prevent ghost fishing	No	N/A	N/A	No	Yes	Yes	Yes
**Overall conservation score**	**11**	**N/A**	**N/A**	**0**	**24**	**11**	**35**

## 4 Discussion

The present study quantifies for the first time the extent of spatial overlap between loggerhead turtles and fishing activity in the Northeast Atlantic. Although the turtle tracking and fishing data were not contemporaneous, around 79% of turtle locations overlapped with fishing, which was similar to other species that have been investigated, such as 83% of olive ridley turtle locations in Pacific Panama (Guzman et al. 2019). This extensive overlap also aligns with high bycatch levels reported by observers. For example, longlines in the Northeast Atlantic catch loggerheads at a rate of 0.152 turtles/1000 hooks (Parra et al. 2023), and broader estimates suggest that tens of thousands of turtles may be caught annually across various gear types (de la Hoz Schilling et al. 2023; Roast et al. 2023). Coastal trawling overlapped the most with turtles, particularly neritic turtles whose coastal foraging grounds coincided with highly fished areas. In these coastal habitats, turtles may also be exposed to additional gear types not captured in our analysis, such as gillnets and small‐scale fisheries (de la Hoz Schilling et al. 2023).

This analysis provides a useful first approximation of turtle‐fishery overlap in the Northeast Atlantic. Future studies could build on this by incorporating temporal dynamics, as turtle distributions and fishing effort are likely to vary seasonally and interannually. In particular, applying the results of predictive modelling frameworks linking telemetry and environmental variables (e.g., Forney et al. 2015; Pikesley et al. 2015) may help provide more comprehensive assessments of bycatch risk may help provide more comprehensive assessments of bycatch risk.

### 4.1 Frameworks and Policies

Although several regional and global frameworks aim to protect sea turtles, there is likely a significant gap between policy and implementation of effective measures. For example, ICCAT's sea turtle recommendation (22–12) mandates at least one gear modification and sets out safe handling practices, but these only apply to ICCAT‐regulated vessels (ICCAT 2022). International agreements like the Convention on Biological Diversity and the Convention on the Conservation of Migratory Species (CMS), alongside subregional turtle‐specific frameworks like the CMS MoU, also promote conservation efforts. The CMS MoU is an intergovernmental agreement that promotes collaborative sea turtle conservation efforts on the Atlantic coast of Africa and obliges members to take bycatch reduction actions (CMS 2023). Despite these frameworks, well‐evidenced measures such as gear modifications and time‐area management (Table 1) appear largely unimplemented. It is also difficult to fully evaluate the effectiveness of existing strategies due to the lack of data available for many countries (Fuentes et al. 2023).

### 4.2 Countries and Fleets

Loggerhead turtles displayed extensive, international movements (Varo‐Cruz et al. 2013; Monzon‐Arguello et al. 2010), highlighting the need for coordinated international conservation efforts. Predicted hotspots of overlap with fishing in the present study matched reported bycatch hotspots (de la Hoz Schilling et al. 2023). Overlap in Cabo Verde likely reflects a particularly high density of turtles commuting to and from nesting sites at this globally significant colony (Marco et al. 2012; Monzon‐Arguello et al. 2010), as well as the fact that individuals were tagged at this location, leading to increased representation of local movements. Overlap in Senegal and Mauritania may reflect more intense fishing pressure, even if turtle density is lower. Foreign fleets, particularly Chinese and Spanish vessels, accounted for a large proportion of fishing effort in the region. Distant water fleets are often linked to adverse environmental impacts and poor legal compliance (Aquino 2023; Alder and Sumaila 2004). Therefore, stronger enforcement of sustainability clauses in these agreements is essential to reduce bycatch (Belhabib, Sumaila, Lam, et al. 2015).

### 4.3 Area‐Based Management

Specific management tools to reduce bycatch include area‐based management, aiming to protect turtles by restricting fishing in fixed areas (Sala and Giakoumi 2018), as well as time‐area management approaches that account for temporal variation in species distribution (O'Keefe et al. 2014). Satellite tracking studies have demonstrated that green turtles in West Africa benefit substantially from existing MPAs, such as João‐Vieira Poilão and Banc d'Arguin (Patrício et al. 2022; Catry et al. 2023). However, the broad and variable ranges over which loggerhead turtles move, and the different behaviours exhibited in them (e.g., migration, reproduction, wintering), may limit the effectiveness of static protected areas. In addition, the low coverage of protected areas in the present study (16.9%) was similar to reports elsewhere: 15% for Mediterranean loggerheads (Abalo‐Morla et al. 2022) and 10% for leatherback turtles in Equatorial Guinea (Garzon et al. 2023). Fishing persisted in many MPAs, which is common globally, as 94% of MPAs permit some form of fishing (Costello and Ballantine 2015). Alarmingly, this includes high turtle density areas identified in the present study, such as Banc d'Arguin (IUCN 2020), and MPAs in Cabo Verde (Fundação Tartaruga 2021). Spatial closures also carry significant socio‐economic costs to the fishing industry, as fisheries are vital for food security and livelihoods in this region (Belhabib, Sumaila, and Pauly 2015). Instead, dynamic ocean management offers a more flexible alternative, using real‐time ocean data to predict turtle presence and guide spatial recommendations for fishers to reduce bycatch (Howell et al. 2008; Pons et al. 2022). However, real‐world applications, such as ‘TurtleWatch’ in the Hawaii longline fishery, have been limited by low fisher compliance (Howell et al. 2015; Siders et al. 2023).

### 4.4 Gear Types

Trawlers and longlines showed extensive spatial overlap with turtles, and regional reports of bycatch indicate consistent turtle interactions with these gears (Zeeberg et al. 2006; Bourjea et al. 2014; Coelho et al. 2015). Limited spatial overlap with purse seines in the present study aligns with the relatively low turtle bycatch reported by Cardona et al. (2025). Trawling exhibited the greatest overlap with turtle locations, and similarly other studies (Alessandro and Antonello 2010; Lewison et al. 2014; Carbonara et al. 2025) have shown that trawl fisheries have high turtle bycatch rates. This raises concern given the high associated mortality (up to 43% of turtles) of turtles by‐caught by trawlers (Parga et al. 2020). However, Cardona et al. (2025) estimate that by far the largest proportion of turtles are caught by longline fisheries, consistent with other reports from Northwest Africa (de la Hoz Schilling et al. 2023; Roast et al. 2023). This likely reflects the distinction between encounter probability and gear‐specific catchability, as although trawling represented the highest fishing effort in the region, longlines deploy thousands of baited hooks across extensive spatial scales, potentially generating higher turtle capture rates per unit effort. Our findings thus highlight areas of elevated encounter probability, rather than relative mortality risk across gear types.

The loggerhead turtle population nesting on Cabo Verde appears to be size‐structured, with larger, neritic individuals occupying shallow coastal waters and smaller, oceanic turtles occurring offshore (Hawkes et al. 2006; Eder et al. 2012; Cardona et al. 2017). This likely influences gear‐specific exposure, as trawls operate predominantly in coastal areas, while longlines and purse seines are concentrated offshore. Consistent with this, Cardona et al. (2025) reported that larger turtles were associated with trawl bycatch and smaller turtles with longlines. Although based on only two individuals, the dive profiles presented here align with such patterns, with coastal turtles diving to the seabed and offshore turtles occupying surface waters (McClellan et al. 2010), illustrating how vertical habitat use may further mediate gear‐specific risk. These patterns suggest that different fisheries may disproportionately affect distinct demographic components of the population.

### 4.5 Gear Modifications

Gear modifications are among the most promising solutions to reduce turtle bycatch as they can be applied across all regions, while also allowing fishing to continue (Senko et al. 2014). For example, turtle excluder devices (TEDs) are grids inserted into trawl nets that allow turtles to escape, reducing bycatch by up to 99% (Brewer et al. 2006), and have been successfully implemented in U.S. shrimp fisheries (Finkbeiner et al. 2011). As trawlers accounted for over half of regional fishing effort and exhibited the greatest overlap with turtles, TEDs could be a key mitigation tool. Despite this, our analysis suggested they are likely only used in two countries. For longlines, using circle hooks (Piovano et al. 2009; Sales et al. 2010), switching bait from squid to fish (Santos et al. 2013), and adjusting fishing depth (Polovina et al. 2003; Swimmer et al. 2017) has been shown to reduce bycatch. While depth modification is challenging for purse seines and trawls (i.e., vessels generally operate at depths where target fish are likely to be caught), longlines can be set deeper to avoid turtles (Polovina et al. 2003; Swimmer et al. 2017). However, mitigation measures that perform well experimentally may not always translate to equivalent real‐world effectiveness at commercial scales (Hilborn et al. 2026). The success of gear modification strategies ultimately will depend on effective implementation, fisher compliance, stakeholder engagement, and broader commercial incentives (Fuentes et al. 2023; Virgili et al. 2024; Garraud et al. 2023). It is also important to note that gear modifications such as TEDs address bycatch but do not mitigate broader ecological impacts of fishing methods, such as the effects of bottom trawling on seabed habitats (Hiddink et al. 2020; Zhang et al. 2024).

### 4.6 Artisanal and Illegal Fishing

Small‐scale and illegal fishing are prevalent in Northwest Africa (Belhabib et al. 2018) but are not currently recorded by GFW. Therefore, the present study likely underestimates fisheries‐turtle overlap, particularly nearshore, and may overemphasise the relative contribution of industrial fleets to overlap. Artisanal fleets, characterised by small, low‐technology boats in coastal areas, are likely much more numerous than industrial fleets in Northwest Africa (Belhabib et al. 2018) and might contribute substantially to bycatch (Martins et al. 2022). Such impacts are likely to be particularly important in nearshore habitats, where small‐scale fisheries overlap with neritic and reproductive life stages of several turtle species (Martins et al. 2022; Moore et al. 2010). However, artisanal fleets are not required to have AIS, which hinders monitoring (Kroodsma et al. 2018; Sequeira et al. 2019). West Africa also faces some of the highest levels of illegal, unreported and unregulated (IUU) fishing globally, with estimates suggesting that total catches may be up to 40% higher than reported landings, reflecting both illegal activities and underreporting (Doumbouya et al. 2017; Agnew et al. 2009). Vessels fishing illegally often switch off or manipulate their AIS (Welch et al. 2022) (Paolo et al. 2024), thus future research should use predictive models (Welch et al. 2022; Young et al. 2006) or novel tracking techniques (Cardiec et al. 2020; Exeter et al. 2021; Weimerskirch et al. 2020), to improve the detection of artisanal and illegal fishing. Furthermore, catch and bycatch reporting is also often incomplete across both artisanal and industrial fisheries, highlighting the need for improved fisheries monitoring and observer coverage (Moore et al. 2021).

### 4.7 Concluding Remarks

The present study reveals a strong spatial overlap between loggerhead turtle distributions and fishing activity in waters adjacent to West Africa, particularly in coastal areas with intense trawling. This region is a global loggerhead turtle hotspot, hosting one of the world's largest nesting populations, and is facing increasing fishing pressure (Belhabib et al. 2018), so improved bycatch mitigation efforts should be a conservation priority. Existing MPAs and policies likely provide insufficient protection relative to the scale of the threat, and in future, more effective conservation will depend on international collaboration, strong enforcement, and policies that balance ecological goals with socio‐economic realities (Lewison et al. 2011; Arlidge et al. 2020; de la Hoz Schilling et al. 2023).

While spatial overlap serves as a valuable proxy for turtle‐fishery interactions, increased observer coverage would also provide valuable data for quantifying bycatch and mortality (Moore et al. 2021; Cardona et al. 2025). Going forward, modelling approaches (e.g., Forney et al. 2015; Pikesley et al. 2015) could help predict turtle presence. Additionally, biologging tags could also incorporate radar sensors to directly detect vessels (Weimerskirch et al. 2020), and activity sensors may help evaluate the behavioural and physiological impacts of fisheries encounters (Chung et al. 2021; Tyson et al. 2017; Fuentes et al. 2023). Finally, this region hosts other megafauna at risk of bycatch (Zeeberg et al. 2006; de la Hoz Schilling et al. 2023), and using biologging to integrate these species into future overlap assessments would support coordinated, multi‐species conservation strategies (Hindell et al. 2020).

## Author Contributions

**Amy Isabelle Bowler:** conceptualization (equal), data curation (equal), formal analysis (equal), investigation (equal), methodology (equal), validation (equal), visualization (equal), writing – original draft (equal), writing – review and editing (equal). **Annette C. Broderick:** conceptualization (equal), funding acquisition (equal), writing – review and editing (equal). **Michael S. Coyne:** data curation (equal), formal analysis (equal), investigation (equal), writing – review and editing (equal). **Matthew H. Godfrey:** conceptualization (equal), investigation (equal), writing – review and editing (equal). **Brendan J. Godley:** conceptualization (equal), formal analysis (equal), funding acquisition (equal), investigation (equal), methodology (equal), project administration (equal), writing – review and editing (equal). **Pedro Lopez‐Suarez:** investigation (equal), project administration (equal), writing – review and editing (equal). **Nuria Varo‐Cruz:** investigation (equal), methodology (equal), project administration (equal), writing – review and editing (equal). **Lucy. A. Hawkes:** data curation (equal), formal analysis (equal), investigation (equal), methodology (equal), project administration (equal), supervision (equal), writing – review and editing (equal).

## Funding

This work was supported by SeaWorld and Busch Gardens Conservation Fund, European Social Fund Plus, Natural Environment Research Council (NE/X502387/1, NER/I/S/2001/00750), Center for Sponsored Coastal Ocean Research (NA04NMF4550391), Marine Conservation Society, Overseas Territories Environment Programme, People's Trust for Endangered Species and British Chelonia Group.

## Conflicts of Interest

The authors declare no conflicts of interest.

## Supporting information


**Table S1:** Evaluation of turtle bycatch mitigation measures from the Conservation Evidence database, including the number of studies, benefits, and limitations associated with each measure.

## Data Availability

Turtle tracking data is available at Movebank, study ID 8355000618: https://www.movebank.org/cms/webapp?gwt_fragment=page=studies,path=study8355000618.
