# Supplementary material for: Assessing Loggerhead Turtle Exposure to Fisheries in Northwest Africa: Predicted Risk and Management Gaps
Source: Ecol Evol. 2026 Jun 1;16(6):e73729. doi: 10.1002/ece3.73729 (PMC13240276; doi:10.1002/ece3.73729)
Supplement: Supplementary file 1 — Table S1: Evaluation of turtle bycatch mitigation measures from the Conservation Evidence database, including the number of studies, benefits, and limitations associated with each measure. [file ECE3-16-e73729-s001.docx]

# Appendix

**Table A1.** Evaluation of turtle bycatch mitigation measures from the Conservation Evidence database, including the number of studies, benefits, and limitations associated with each measure.

| **Bycatch mitigation measure** | **Number of studies** | **Benefits** | **Limitations** |
| --- | --- | --- | --- |
| **Use circle hooks instead of J hooks** | 11 | Reduces bycatch and increases post-release survivorship by reducing the likelihood of turtles becoming hooked or swallowing hooks (Sales et al., 2010) | May affect target catch for some species (Sales et al., 2010; Read, 2007) |
| **Use a different bait type** | 9 | Using fish instead of squid bait reduces unwanted turtle catch as turtles are less attracted to it (Santos et al., 2013) | Mixed effects on target catch (Gilman et al., 2007), and may increase bycatch of other species (Gilman et al., 2016) |
| **Exclusion and escape devices on trawl gear (“Turtle Excluder Devices”)** | 6 | Allows turtles to escape from trawl nets if caught (Sala et al., 2011) | Catch rates of target species vary depending on device design (Robins-Troeger, 1994); do not mitigate broader ecological impacts of trawling (Hiddink et al., 2020; Zhang et al., 2024) |
| **Add lights to fishing gear** | 5 | Decreases turtle catch rates in gillnets while maintaining target catch (Allman et al., 2021) | Effectiveness varies with environmental factors (Allman et al., 2021), and some studies suggest turtles move towards lights (Wang et al., 2007) |
| **Deploy fishing gear at different depths** | 3 | Reduces interactions with turtles by keeping fishing gear outside their foraging range (Peckham et al., 2016) | Effectiveness depends on the species, and may reduce target catch of epipelagic species (Beverly et al., 2009; Swimmer et al., 2017) |
| **Temporary fishery closures** | 3 | Reduces the risk of turtles being caught during the closure period (Lewison et al., 2003) | Limited effectiveness if enforcement is weak or if fishing resumes quickly after closures (Lewison et al., 2003) |
| **Reduce duration of time fishing gear is in the water** | 2 | Reduces mortality rates by decreasing submergence time when turtles are caught (Echwikhi et al., 2012) | Minimal effect on the number of turtles caught (Swimmer et al., 2017) |
| **Visual deterrents on fishing gear** | 2 | Predator shapes delay turtle approach and reduce bycatch (Wang et al., 2010) | Reduces target catch (Wang et al., 2010) |
| **Use larger hooks** | 2 | Turtles are less likely to be caught and less likely to swallow hooks (Stokes et al., 2011; Parga et al., 2015) | Reduces target catch, and may be ineffective at preventing the capture of larger turtles (Stokes et al., 2011) |
| **Use dyed bait** | 2 | May reduce attractiveness of bait to sea turtles (Swimmer et al., 2005) | Preference varied depending on species, and similar turtle capture rates overall (Swimmer et al., 2005) |
| **Implement turtle bycatch quotas** | 1 | Reduces pressure on populations, allowing time for recovery (Howell et al., 2008) | “Race to fish” may be incentivised, resulting in minimal bycatch avoidance  (Siders et al., 2023) |
| **Promote knowledge exchange between fishers to improve good practice** | 1 | Helps promote and share conservation information, for example to promote the avoidance of turtles (Howell et al., 2008) | Following introduction of a knowledge exchange tool, bycatch was similar; fishers did not follow recommendations (Howell et al., 2008) |
| **Use non-ringed hooks** | 1 | Fewer turtles caught when using non-ringed hooks (Piovano & Swimmer, 2017) | Reduces target catch (Piovano & Swimmer, 2017) |
| **Change hook baiting technique** | 1 | Single baiting reduces the risk of turtles swallowing hooks as it is easier to remove bait (Stokes et al., 2011) | Research limited to laboratory settings and may lead to higher bait loss, impacting fishing efficiency (Stokes et al., 2011) |
